# Does Treatment of Adolescent Fractures Differ between Specialties? A Survey among Pediatric and Trauma Surgeons

**DOI:** 10.3390/jpm14080842

**Published:** 2024-08-09

**Authors:** Alexander Hanke, Eva Scheerer-Harbauer, Christian Wulbrand, Clemens Memmel

**Affiliations:** 1 Department of Pediatric Surgery and Pediatric Orthopedics, Clinic St. Hedwig, Barmherzige Brueder Regensburg, KUNO Pediatric University Medical Center, 93049 Regensburg, Germany; alexander.hanke@barmherzige-regensburg.de (A.H.);; 2 Department of Trauma Surgery, Orthopedics and Sports Medicine, Barmherzige Brueder Regensburg, 93049 Regensburg, Germany; 3 Department of Trauma Surgery, University Medical Center Regensburg, 93053 Regensburg, Germany

**Keywords:** adolescent fracture, pediatric traumatology, trauma surgery, standardized treatment

## Abstract

From a traumatological point of view, adolescents (12–18 years) represent a special group of patients. This is due to their biomechanical characteristics being between pediatric and adult fracture types. In Germany, they are treated by both pediatric and trauma surgeons. For this survey, seven cases of adolescent fractures were evaluated by both pediatric and trauma surgeons and their preferred treatment options were raised. The questionnaires were completed anonymously. Additionally, information on the specialty and years of experience were asked. In total, 126 valid questionnaires were obtained (from 78 pediatric and 48 trauma surgeons). The respondents’ mean clinical experience was high (71.5% stated more than 10 years of surgical experience). For every single exemplary case, a significant difference in therapy decisions between the groups could be found. For the demonstrated seven cases, a tendency toward more operative and more invasive treatments was found with trauma surgeons compared to pediatric surgeons. On the other hand, there was a risk of underestimating the severity of fracture entities similar to adult fractures in pediatric surgeons. Overall, a continuous interdisciplinary exchange between both surgical specialties is necessary to ensure optimal treatment for adolescent fractures and to develop guidelines in the future.

## 1. Introduction

In many countries, e.g., Germany, fractures in adolescents (12–18 years) are treated by either pediatric or trauma surgeons, depending on regional or hospital policy. This is not only due to the rescue service system but also to often significant differences between the nominal age of the patient and the skeletal maturity of the patient. In adolescents, fracture types can be found that belong to adults due to the beginning or completion epiphyseal closure.

For pediatric fractures, reference literature [1,2] and guidelines [3,4,5] exist. Furthermore, extensive literature and guidelines for adult and geriatric trauma can be found. However, except for femoral fractures [6], there are no guidelines specifically for adolescent fractures in Germany. These fractures are extremely challenging to treat as age, physeal status, corrective potential, height, and weight have to be considered and often do not correlate [7].

A recent survey showed that the application of X-ray and aftercare regimens of pediatric and adolescent fractures differ significantly [8,9]. In line with this finding, the following survey was conducted to evaluate whether the treatment strategy of adolescent fractures also differs depending on the specialty of the treating physician. The overall aim of this research project is to develop standardized treatment concepts for adolescent fractures, irrespective of the specialty of pediatric or trauma surgery. This pilot study represents the first step in this direction, as it is intends to provide a descriptive representation of current differences in the treatment of adolescent fractures.

## 2. Materials and Methods

**Figure 1:**
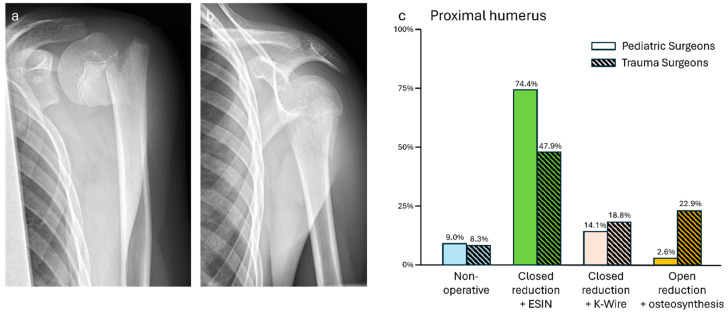
(**a**) Anterior–posterior and (**b**) lateral X-rays of a proximal subcapital humeral fracture (f, 13 y, 150 cm, 48 kg). (**c**) Descriptive data of the survey results, indicated in percentages.

**Figure 2:**
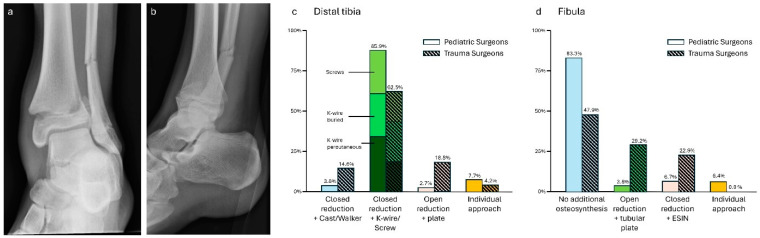
(**a**) Anterior–posterior and (**b**) lateral X-rays of a distal lower leg fracture (m, 15 y, 170 cm, 60 kg). Descriptive data of the survey results for the (**c**) distal tibia and (**d**) fibula, indicated in percentages.

**Figure 3:**
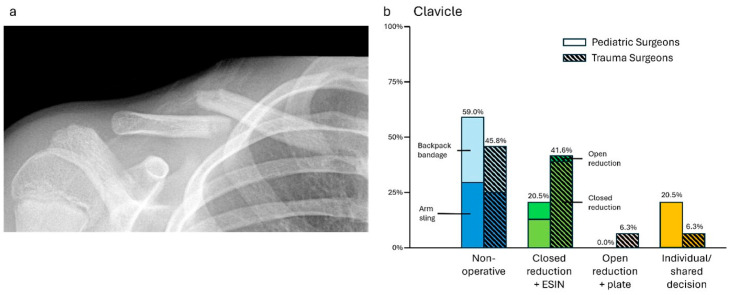
(**a**) Anterior–posterior X-ray of a dislocated clavicle shaft fracture (m, 13 y, 160 cm, 40 kg). (**b**) Descriptive data of the survey results, indicated in percentages.

**Figure 4:**
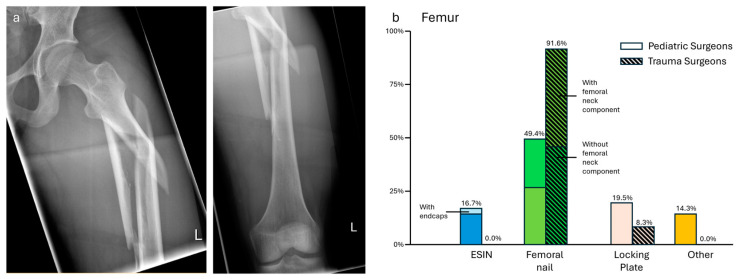
(**a**) X-ray of a multifragmentary femoral fracture (f, 17 y, 165 cm, 55 kg). (**b**) Descriptive data of the survey results, indicated in percentage.

**Figure 5:**
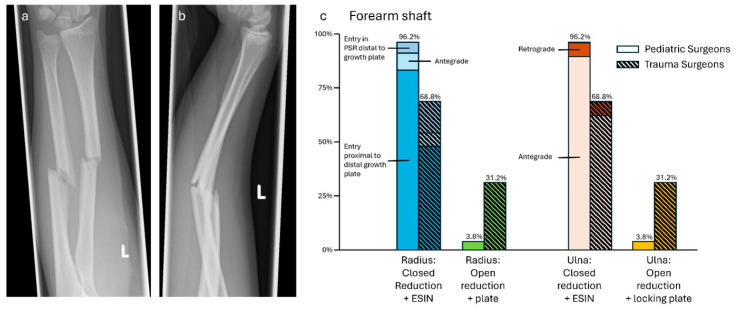
(**a**) Anterior–posterior and (**b**) lateral X-rays of a dislocated forearm shaft fracture (f, 14 y, 155 cm, 45 kg). (**c**) Descriptive data of the survey results, indicated in percentages.

**Figure 6:**
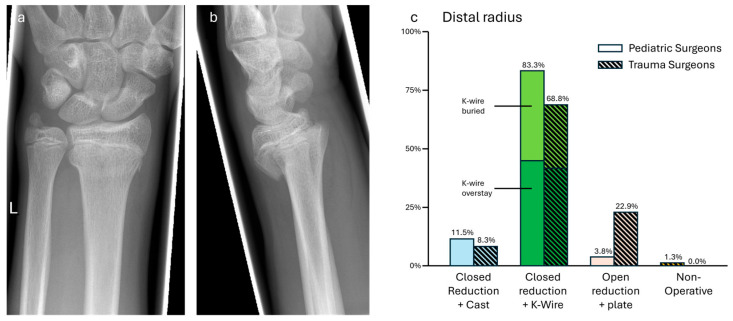
(**a**) Anterior–posterior and (**b**) lateral X-rays of a distal radial fracture with concomitant non-dislocated fracture of the styloid process of the distal ulna (m, 17 y, 185 cm, 85 kg). (**c**) Descriptive data of the survey results, indicated in percentages.

**Figure 7:**
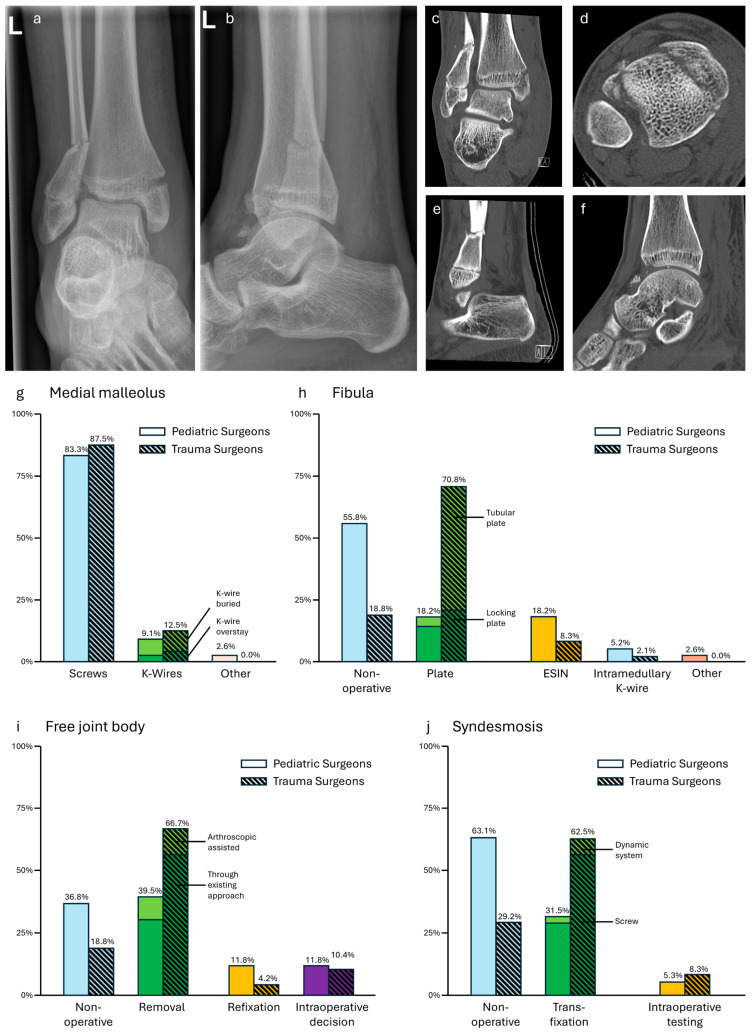
(**a**,**b**) X-rays and (**c**–**f**) representative CT layers of a bimalleolar fracture with concomitant a Tillaux fracture and free joint body (m, 14 y, 175 cm, 55 kg). (**g**–**j**) Descriptive data of the survey results, indicated in percentages.

This study presents the results of a survey in the form of an online questionnaire, which was sent to German departments of pediatric and trauma surgery. For the questionnaire, X-ray images of adolescent fractures (age 12–18) in past years were screened. Seven cases were chosen by the research team, which were deemed fitting for the questionnaire. These cases were as follows, by order of appearance in the questionnaire:

1. Displaced subcapital humeral Salter–Harris Type 2 fracture (growth plate open, f, 13 y, 150 cm, 48 kg; Figure 1);
2. Distal tibia Salter–Harris Type 2 fracture with concomitant distal fibula shaft fracture (growth plate still visible, m, 15 y, 170 cm, 60 kg; Figure 2);
3. Midshaft clavicle fracture (proximal humeral growth plate open, m, 13, 160 cm, 40 kg; Figure 3);
4. Mulifragmentary femoral shaft fracture in the proximal third (growth plates closed, f, 17 y, 165 cm, 55 kg; Figure 4);
5. Transverse forearm shaft fracture (growth plates almost closed, f, 14 y, 155 cm, 45 kg; Figure 5);
6. Dorsally displaced Salter–Harris Type 2 distal radius fracture (growth plates still visible, m, 17 y, 185 cm, 85 kg; Figure 6);
7. Bimalleolar fracture with additional Tillaux fragment and loose joint body (growth plates open, m, 14, 175 cm, 55 kg; Figure 7).

In addition, information on age, weight, and height was provided, as this must be included in the decision-making process for the preferred therapy. Different treatment options were given as choices for every fracture; furthermore, a field for free input was provided if the treatment of choice was not listed. An option for respondents’ comments existed for every case. The questionnaire finished with general questions about the primarily responsible department for adolescent fractures and the frequency of pediatric, adolescent, adult, and geriatric fractures in daily practice. Furthermore, the respondents’ specialty and their years of experience were evaluated. The questionnaire was completely anonymous for the shown X-rays and the respondents’ data. The materials and methods were checked and approved by the institutional review board and the local ethics committee of the University of Regensburg before launching the survey (protocol code: 24-3804-180; date of approval: 22 December 2023).

The survey was conducted with a free online survey tool (SoSci Survey GmbH, Munich, Germany). The link to the survey was open and sent to all pediatric surgical departments in Germany (85 clinics) and the corresponding departments for trauma surgery in the same clinic with the bid to share the questionnaire with the whole team. Furthermore, the link was shared with the members of the German association of pediatric surgery (DGKCH) and the German young forum for orthopedics and trauma surgery (JFOU). Reminders were sent two weeks later. The survey was accessible for two months (April and May 2024). In addition to fully completed questionnaires, a further inclusion criterion was the respondent’s membership of a pediatric surgery or trauma surgery department with a certain level of clinical experience in the treatment of adolescent fractures. Incomplete questionnaires, especially with no information on the specialty, were excluded.

The evaluation and statistical analysis were conducted with SPSS software package (Version 24.0, IBM SPSS Inc., Chicago, IL, USA). Differences between the groups were tested by Pearson’s chi-squared test. The level of significance was set to *p* = 0.05.

## 3. Results

Two clinics for pediatric surgery declined participation, because they stated they were not involved in pediatric or adolescent trauma. The link to the questionnaire was clicked 339 times. A total of 144 participated in the survey, although 18 did not finish it. This resulted in 126 questionnaires suitable for analysis. Of those, 71 were pediatric surgeons (“ped”) and 48 trauma surgeons (“trauma”). A total of seven surgeons who were specialized in both were exclusively treating children and adolescents, thus their results were added to the “ped” group.

A total of 31.0% (39/126) reported more than 20 years of surgical experience, 40.5% (51/126) between 10 and 20 years, 15.9% (20/126) between 5 and 10 years, and 12.7% (16/126) less than 5 years. This was similar in both groups (*p* = 0.56).

The cut-off value for the treatment of adolescent trauma by pediatric surgeons was primarily the patient’s 18th birthday (95/126, 75.4%). Furthermore, the 16th (10/126, 7.9%) and 15th (5/126, 4.0%) birthday and, in two cases, the 14th and 10th birthday were named. A total of 11.1% (14/126) reported a general interdisciplinary approach to adolescent trauma. No significant difference between either group regarding cut-off values was found (*p* = 0.08).

As expected, the reported frequency of pediatric trauma (0–12 years) more than once a week was significantly higher for pediatric surgeons (*p* < 0.01). On the other hand, adult and geriatric trauma was treated almost exclusively by trauma surgeons (*p* < 0.01). Adolescent trauma (12–18 years) was treated more often on a daily to weekly basis by pediatric surgeons (ped: 61/78, 78.2%; trauma: 23/48, 47.9%; *p* < 0.01) (Table 1).

A statistical effect between the frequency of treating adolescent fractures and the treatment strategy could only be found for the forearm fracture in case 5 (radius: *p* = 0.03, ulna: *p* < 0.01) and the fibula fracture in case 7 (*p* = 0.03). In these named cases, the treatment option of elastic stable intramedullary nailing (ESIN) was preferred to plate osteosynthesis the more often adolescent trauma occurred in the daily practice. For all other cases and questions, no significant effect was found (*p* > 0.05, respectively).

### 3.1. Case 1—Proximal Humeral Fracture

Closed reduction and ESIN osteosynthesis were the favored options for both pediatric (58/78, 74.4%) and trauma surgeons (23/48, 47.9%). Closed reduction and percutaneous K-Wires were the second most frequent options (ped: 11/78, 14.1%; trauma: 9/48, 18.8%). Non-operative treatment was named 11 times (ped: 7/78, 9.0%; trauma: 4/48, 8.3%). Primary open reduction was named mainly by trauma surgeons (ped: 2/78, 2.6%; trauma: 11/48, 22.9%). For osteosynthesis after open reduction, besides ESIN and K-wires, locking plates (twice) and cannulated screws (once) were named by trauma surgeons. Finally, one trauma surgeon proposed closed reduction and then conservative treatment. The differences in the treatment strategies between both specialties were significant (*p* = 0.01, see Figure 1).

### 3.2. Case 2—Distal Lower Leg Fracture

The three frequently named options for treatment of the distal tibia fracture were similar in approach (ped: 67/78, 85.9%; trauma: 30/48, 62.5%): closed reduction and retention either with overstaying (ped: 27/78, 34.6%; trauma: 9/48, 18.8%), buried K-wires (ped: 21/78, 26.7%; trauma: 12/48, 25.0%), or percutaneous screws (ped: 19/78, 24.4%; trauma: 9/48, 18.8%). Both closed reduction and no further osteosynthesis (ped: 3/78, 3.8%; trauma: 7/48, 14.6%) or osteosynthesis with plates (ped: 2/78, 2.6%; trauma: 9/48, 18.8%) were more often suggested by trauma surgeons. Among individual approaches (ped: 6/78, 7.7%; trauma: 2/48, 4.2%), three pediatric surgeons opted for open reduction to remove interfragmentary periost followed by K-wire fixation. An additional preoperative computed tomography (CT) was requested by one pediatric and one trauma surgeon.

No additional treatment of the fibula and immobilization by cast or walker was suggested by most of the surgeons (ped: 65/78, 83.3%, trauma: 23/48, 47.9%). As a rationale, indirect reduction via the interosseous membrane was additionally given by some participants. Nevertheless, osteosynthesis of the fibula by either tubular plates (ped: 3/78, 3.8%; trauma 14/48, 29.2%) or ESIN (ped: 5/78, 6.4%; trauma: 11/48, 22.9%) was more often named by trauma surgeons. Four pediatric surgeons (4/78, 5.1%) opted for an individual approach due to intraoperative stability testing and one opted for open reduction and locking plate osteosynthesis (1/78, 1.3%).

The treatment of the distal tibia and the distal fibula showed significant differences between the two groups (*p* < 0.01, respectively, see Figure 2).

### 3.3. Case 3—Clavicle Fracture

Non-operative treatment (ped: 46/78, 59.0%; trauma: 22/48, 45.8%), by either arm sling (ped: 23/78, 29.5%; trauma: 12/48, 25%) or backpack bandage (ped: 23/78, 29.5%; trauma: 10/48, 20.1%), was favored by both groups. Nevertheless, operative treatment by closed reduction and ESIN was chosen more frequently by trauma surgeons (ped: 10/78, 12.8%; trauma: 18/48, 37.5%). Less often named was open reduction and ESIN (ped: 6/78, 7.7%; trauma: 2/48, 2.6%) or plating, which was only chosen by trauma surgeons (3/48, 6.3%). It has to be mentioned that, in particular, pediatric surgeons opted to promote both operative and non-operative as equal options to the parents. Furthermore, it was added by the participants in the free text box that the decision should be made based on individual factors, especially the patient’s sport demand (ped: 16/78, 20.5%; trauma 3/48, 6.3%). Although conservative treatment was the most common choice for both specialties, more trauma surgeons tended to favor surgical treatment (*p* < 0.01, see Figure 3).

### 3.4. Case 4—Multifragmentary Femoral Fracture

All surgeons saw the need for operative treatment. For patient positioning and reduction of the fracture, both pediatric and trauma surgeons preferred a fracture table (ped: 62/73, 84.9%; trauma: 37/48, 77.1%) over a surgical table (ped: 11/73, 15.1%; trauma: 11/48, 22.9%). No significant difference in the patient positioning was found (*p* = 0.08).

Osteosynthesis antegrade nailing, with or without the femoral neck component, was the treatment of choice, although trauma surgeons were much more in favor (ped: 38/77, 49.4%; trauma: 44/48, 91.6%). Except for locking plates (ped: 15/77, 19.5%; trauma: 4/48, 8.3%), trauma surgeons did not choose any other option. Pediatric surgeons also opted for ESIN without endcaps (11/77, 14.3%) and with endcaps (2/77, 2.6%). Other options were external fixation as the definitive treatment (4/77, 5.2%) and retrograde nailing (3/77, 3.9%). Four pediatric surgeons (4/77, 5.2%) noted that they would refer such a fracture type to the trauma department. This difference in surgical approaches was significant (*p* < 0.01, see Figure 4). One pediatric surgeon did not give an answer to this case.

### 3.5. Case 5—Forearm Shaft Fracture

All surgeons saw the need for surgical treatment. Both specialties preferred osteosynthesis with ESIN. To address the fracture of the radius, a retrograde ESIN with entry point proximal to the growth plate was the treatment of choice (ped: 65/78, 83.3%; trauma: 23/48, 47.9%). Retrograde ESIN with an entry point distal to the growth plate (ped: 4/78, 5.1%; trauma: 7/48, 14.6%) or ESIN in antegrade fashion (ped: 6/78, 7.7%; trauma: 3/48, 6.3%) were also named. The need for locking plating was seen more by trauma surgeons (ped: 3/78, 3.8%; trauma: 15/48, 31.3%). The treatment of the ulna, in analogy to the radius, occurred mainly with ESIN in antegrade fashion (ped: 70/78, 89.7%; trauma: 30/48, 62.5%) and sometimes in retrograde fashion (ped: 5/78, 6.4%; trauma: 3/48, 6.3%). Locking plates were preferred by the same surgeons that opted for locking plating of the radius (ped: 3/78, 3.8%; trauma: 15/48, 31.3%). No hybrid option (i.e., combination of ESIN and plates) was promoted. Trauma surgeons were more likely to favor locking plates than pediatric surgeons. (*p* < 0.01, respectively, see Figure 5). It must be noted that three trauma surgeons added the information that a non-locking plate would also be sufficient (3/15, 20.0%).

### 3.6. Case 6—Distal Radius Fracture

Closed reduction and percutaneous K-wiring was the treatment of choice (ped: 65/78, 83.3; trauma: 33/48, 68.8%). Leaving the K-wires overstay was mentioned slightly more often (ped: 35/78, 44.9%; 20/48, 41.7%) than burying them (ped: 30/78, 38.5%; 13/48, 27.1%). While closed reduction and casting was the second choice of pediatric surgeons (ped: 9/78, 11.5%; trauma: 4/48, 8.3%), open reduction and plating was the second choice for trauma surgeons (ped: 3/78, 3.8%; trauma: 11/48, 22.9%). Complete non-operative treatment was only named by one pediatric surgeon. The more frequent choice of plating among trauma surgeons was significant (*p* = 0.02, see Figure 6).

### 3.7. Case 7—Bimalleolar Fracture

For the treatment of the medial malleolus, screws were the treatment of choice for both specialties (ped: 68/77, 88.3%; trauma: 42/48, 87.5%). K-wires were proposed by some participants (ped: 7/77, 9.1%; trauma: 6/48, 12.5%). One pediatric surgeon opted for non-operative treatment and one for plating. Overall, no significant difference was found (*p* = 0.81, see Figure 7).

In contrast to this, the fibula was mainly treated non-operative by pediatric surgeons (ped: 43/77, 55.8%, trauma: 9/48, 18.8%), while trauma surgeons preferred tubular plating (ped: 11/77, 14.3%; trauma: 24/48, 50.0%). ESIN (ped: 14/77, 18.2%; trauma: 4/48, 8.3%), intramedullary K-wire (ped: 4/77, 5.2%; trauma: 1/48, 2.1%), and locking plates (ped: 3/77, 3.9%; trauma: 10/48, 20.8%) were also named. Two pediatric surgeons opted for intraoperative stability testing of the fibula before making a treatment decision. This variance between the treatment options had a significant difference (*p* < 0.01, see Figure 7).

Screw fixation of the Tillaux fragment was mainly chosen by both groups (ped: 51/76, 67.1%; trauma: 40/48, 83.3%). Although non-operative treatment was higher with pediatric surgeons (ped: 17/76, 22.4%; trauma: 4/48, 8.3%), there was no significant difference in this treatment option (*p* = 0.17).

Trauma surgeons mainly recommended removing the free joint body through an arthrotomy of existing approaches on the medial or laterals side (ped: 23/76, 30.3%, trauma: 27/48, 56.3%). Pediatric surgeons mainly favored leaving it (ped: 28/76, 36.8%; trauma: 9/48, 18.8%). Other options like arthroscopic removal (ped: 7/76, 9.2%; trauma: 5/48, 10.4%) or refixation (ped: 9/76, 11.8%; trauma: 2/48, 4.2%) were chosen. An individual approach with intraoperative decision was promoted by the free answer option (ped: 9/76, 11.8%; trauma: 5/48, 10.4%). The differences in the approach for the free joint body were significant (*p* = 0.04, see Figure 7).

While pediatric surgeons mainly did not choose special treatment of the syndesmosis (ped: 48/76, 63.1%; trauma: 14/48, 29.2%), trauma surgeons preferred a syndesmotic screw transfixation (ped: 22/76, 28.9%; trauma: 27/48, 56.3%). It has to be noted that, besides a few votes for dynamic syndesmotic systems (ped: 2/76, 2.6%; trauma: 3/48, 6.3%), some pediatric and trauma surgeons recommended intraoperative testing after refixation of the Tillaux fragment (ped: 4/76, 5.3%; trauma: 4/48, 8.3%). Nevertheless, the difference between both groups was significant (*p* < 0.01, see Figure 7).

Finally, most trauma surgeons proposed the usage of an intraoperative CT scan (ped: 11/77, 14.3%; trauma: 31/48, 64.6%), while pediatric surgeons did not (ped: 30/77, 39.0%; trauma: 10/48, 20.8) or did not have the equipment to do so (ped: 25/77, 32.5%; trauma: 6/48, 12.5). Some pediatric surgeons would obtain a postoperative CT scan (ped: 11/77, 14.3%; trauma: 1/48, 2.1%). This difference in the approach to intra- and postoperative CT imaging was significant (*p* < 0.01). 

Two pediatric surgeons did not give answers to all questions associated with this case.

## 4. Discussion

This study reports therapy decisions made by pediatric and trauma surgeons in an online survey regarding seven different adolescent fractures. As the main result, the survey clearly states that in every single case, the given therapy decisions significantly depend on the physician’s specialty. For the demonstrated seven cases, a tendency toward more operative and more invasive treatments was found for trauma surgeons compared to pediatric surgeons. On the other hand, there was a risk of underestimating the severity of fracture entities similar to adult fractures in pediatric surgeons. In the following, we discuss the results for each underlying case.

Proximal humeral fracture—Proximal humeral fractures in adolescents are known to be mainly Salter–Harris Type 2. Surgery is recommended if there is more than >20° angulation and more than one-third shaft width displacement because of the reduced remodeling potential with >13-year-olds [10,11]. With an angulation of more than 45° and full displacement of the shaft in the shown X-ray, operative treatment would be advisable. This was recommended in consensus by more than 90% of both pediatric and trauma surgeons. Although closed reduction and retrograde ESINs are recommended [11,12], more trauma surgeons tended to use more invasive procedures. Nevertheless, independent of the treatment, subcapital fractures in adolescents are described to have a good outcome [13].

Distal lower leg fracture—A survey among 37 pediatric orthopedic surgeons by Swarup et al. [14] showed significant differences in the treatment of Salter–Harris Type 2 distal tibia fractures in children and adolescents. Although closed reduction and casting can be successful, it has a known risk of failure [15] and was not the treatment of choice for both pediatric and trauma surgeons in this survey. Closed reduction and K-wires or screws were the most suggested. Therefore, trauma surgeons were more likely to use plate osteosynthesis than pediatric surgeons. Greater differences were seen in the treatment of the concomitant fibula fracture. A total of 52.1% of trauma surgeons proposed osteosynthesis in accordance with the literature for adult fractures [16]. On the other hand, Wang et al. demonstrated that additional fixation of the fibula in children (mean age 7.8 years) was not beneficial compared to conservative management [17]. This is in line with the majority opinion (83.3%) of pediatric surgeons. Therefore, in our opinion, factors like the patient’s weight have to be taken into consideration, to see if additional stability is needed.

Clavicle fracture—Luo et al. [18] found that adult trauma surgeons were more likely to operate on adolescent clavicle fractures than pediatric orthopedics (32.6% vs. 10.3%) in their institution. This is in accordance with this study’s results (trauma: 41.6% vs. ped: 20.5%). Furthermore, the existing literature does not offer a guideline for the indication for non-operative or operative treatment [19,20]. Even the choice between ESIN and plate osteosynthesis is not conclusively discussed, although there is evidence that even multifragmentary clavicle fractures can be treated well with intramedullary osteosynthesis [21]. All in all, not only the fracture pattern but also individual risk factors and activity levels must be considered for making treatment decisions [22].

Multifragmentary femoral fracture—This case showed a scenario of an adolescent with adult bone status and adult fracture type. While both specialties had the same approach to reduction, there were significant differences in the mode of fixation. Almost all trauma surgeons opted for antegrade femoral nailing according to the AO Principles [23] and national guidelines [6]. It should be noted that if the physis is still open, special nail designs with a lateral entry point should be used [24]. Pediatric surgeons gave a broad range of treatment options, which were in analogy to the treatment of pediatric trauma. Although ESINs are a valid treatment option for adolescent femoral fractures, the fracture pattern and weight have to be taken into consideration [25,26]. Locking plates, external fixation, and retrograde nails might also be options, but offer less postoperative weight bearing compared to intramedullary nailing. It has to be noted that some pediatric surgeons explicitly stated that they would refer such an adult fracture pattern to trauma surgeons.

Forearm shaft fracture—ESINs are the recommended treatment for forearm shaft fractures in children [27,28]. In adolescence, the decision between ESIN and plating is difficult, as Ho et al. described for radius shaft fractures, as no predictive parameters exist for the need for stability and remodeling potential exists to some extent [29]. This can be transferred to forearm shaft fractures. In this survey, both specialties preferred ESINs. Nevertheless, the results show that trauma surgeons tend more towards plating than pediatric surgeons. It is noteworthy that although hybrid fixation with ESIN and plate [30] is a described and known technique for adolescent forearm shaft fractures, neither pediatric nor trauma surgeons opted for such treatment.

Distal radius fracture—Salter–Harris Type 2 fractures at the distal radius are very common in adolescents [31]. Instead of the initial degree of displacement, age was found as the main factor for loss of reduction after closed reduction and casting, which occurred in about 30% [32]. A review by Greig et al. [33] found that up to 15° of displacement is tolerable in adolescents and recommended K-wire fixation and a cast as the operative treatment, over plating. Nevertheless, it has to mentioned that plating offers the option of direct anatomical reduction and cast-free aftercare.

Bimalleolar fracture—The Tillaux fracture is described as a typical adolescent fracture. Because of its intra-articular component, surgical treatment is recommended when displacement is >2 mm [34,35]. It is still under discussion if closed or open reduction is superior [36]. The shown example had a concomitant bimalleolar fracture making it a rare but well-described type of adolescent fracture [37,38,39]. Pediatric and trauma surgeons showed similar survey results: both the Tillaux fracture and the medial malleolus needed screw fixation. More pediatric surgeons addressed the syndesmosis with refixation of the Tillaux fragment and saw no need for osteosynthesis of the fibula, according to pediatric trauma [40]. On the other hand, trauma surgeons opted for additional fixation of the fibula, in line with adult trauma [23]. Therefore, mainly syndesmotic screw fixation was named, although new dynamic techniques might be superior [41]. Removal of the free joint body was more often proposed by trauma surgeons. As they opted for more invasive osteosynthesis and, thus, bigger surgical approaches, it can be assumed that envisioning the ankle joint is easier for them and they have more familiarity with open joint surgery. The anatomical reduction of the syndesmosis is vital for a good clinical outcome. The advantages of intraoperative (cone beam) CT imaging in adult ankle fractures with syndesmotic injuries provides an anatomical reduction in the syndesmosis [41,42]. Nevertheless, no data for this type of intraoperative diagnostic exist for pediatric and adolescent ankle fractures. The survey showed not only significant differences in the availability of intraoperative (cone beam) CT imaging between pediatric and trauma surgeons but also in their application. It can be assumed that pediatric surgeons keep more strictly to the ‘as low as reasonably achievable’ principle (ALARA) for X-ray [9].

The cases of the survey show clearly that the recommended treatment is highly dependent on the physicians’ affiliation to the respective specialty—in this case, pediatric or trauma surgery. First, one reason for the different treatment decisions may be the simple fact that preference is given to procedures that are familiar and frequently used. This bias, of course, applies for both specialties, as it could be seen for the forearm fracture (case 5) and the distal fibula fracture of the last case (case 7). In addition, the lack of clear guidelines for most adolescent fractures leads to disagreement in their care in everyday clinical practice. Even though the patient’s outcome of the hypothetical treatments, unfortunately, cannot be recorded via this study, it must be assumed that such a broad range of treatment recommendations cannot be beneficial for the outcome in general. It is important to note that each treatment decision for adolescent fractures should be made on an individual and multifactorial basis but while following standardized recommendations. Validated guidelines have proven to be the best way to ensure an ideal outcome for each patient. The result of this survey, therefore, shows the need for an interdisciplinary approach for establishing guidelines for adolescent fracture types.

Despite the high response rate of experienced surgeons, the return rate of resident surgeons was rather low. The reason for this effect remains unclear. Nevertheless, in clinical practice, it is the experienced specialist physician who is entitled to make the indication for conservative surgical treatment and who chooses the type of osteosynthesis. The distribution of the respondents, therefore, reflects the everyday clinical practice more closely. Nevertheless, the survey was sent to all clinics with a pediatric surgery department and the corresponding trauma surgery departments. Usually, these are major clinics. It can be assumed that no or, by chance, only a few results were obtained from smaller trauma surgery departments that usually treat both pediatric and adolescent trauma. Although the seven cases were chosen with care, many other adolescent fracture types exist, and the results cannot be generalized. More cases would have made the survey longer and less practicable. The most common treatment options from non-operative to maximally invasive were given to be chosen from. Although a free input field for other treatment options was offered, the bias presented by these pre-given answers cannot be fully excluded. This survey does not provide information about the outcome of the hypothetic therapy decision of the respondents. This marks a limitation within the discussion of our results, as there might be more than one adequate decision. The lack of further information on comorbidities, the patients’ activity level, or similar factors means that the surgeon’s decision is hypothetical and not all factors influencing the therapy decision were named. Nevertheless, the questionnaire was suitable for answering the research question.

## 5. Conclusions

The treatment of adolescent fractures depends, to some extent, on the specialty of the treating surgeon. For the demonstrated seven cases, a tendency towards more operative and more invasive treatment was found for trauma surgeons compared to pediatric surgeons. There was a risk of underestimating the severity of fracture entities similar to adult fractures in pediatric surgeons. In conclusion, this survey calls for continuous interdisciplinary exchange between both surgical specialties to ensure optimal treatment of adolescent fractures and to develop guidelines for the future.

## Figures and Tables

**Table 1:** Reported frequency in treatment of different age groups, sorted by specialty; ped = pediatric surgeons (*n* = 78); trauma = trauma surgeons (*n* = 48). Field coloring marks percentage of number of participants: blank: <12.5%, shaded light green: 12.5% to 25.0%, medium: 25.0% to 50%, dark green: >50% of the group.

		Never	1x/mon.	1x/week	>1x/week	Daily
children (0–12 y)	ped (*n*)	3	0	2	21	52
	trauma (*n*)	8	9	9	7	15
adolescents (12–18 y)	ped (*n*)	2	3	12	27	34
	trauma (*n*)	1	10	14	11	12
adults (>18 y)	ped (*n*)	67	7	0	0	4
	trauma (*n*)	6	3	2	8	29
geriatrics (>75 y)	ped (*n*)	74	1	1	0	2
	trauma (*n*)	10	0	1	5	32

## Data Availability

The data that support the findings of this study are available on request from the corresponding author (C.M.). The data are not publicly available due to restrictions, as the information they contain could compromise the privacy of the research participants.
